# Probucol alleviates atherosclerosis and improves high density lipoprotein function

**DOI:** 10.1186/1476-511X-10-210

**Published:** 2011-11-12

**Authors:** Jian-Kai Zhong, Zhi-Gang Guo, Chen Li, Zhen-Kun Wang, Wen-Yan Lai, Yan Tu

**Affiliations:** 1 Division of Cardiology, Nanfang Hospital, Southern Medical University, Guangzhou 510515, Guangdong, P.R. China

**Keywords:** probucol, high density lipoprotein function, reverse cholesterol transport, Paraoxonase 1, Myeloperoxidase

## Abstract

**Background:**

Probucol is a unique hypolipidemic agent that decreases high density lipoprotein cholesterol (HDL-C). However, it is not definite that whether probucol hinders the progression of atherosclerosis by improving HDL function.

**Methods:**

Eighteen New Zealand White rabbits were randomly divided into the control, atherosclerosis and probucol groups. Control group were fed a regular diet; the atherosclerosis group received a high fat diet, and the probucol group received the high fat diet plus probucol. Hepatocytes and peritoneal macrophages were isolated for [^3^H] labeled cholesterol efflux rates and expression of ABCA1 and SR-B1 at gene and protein levels; venous blood was collected for serum paraoxonase 1, myeloperoxidase activity and lipid analysis. Aorta were prepared for morphologic and immunohistochemical analysis after 12 weeks.

**Results:**

Compared to the atherosclerosis group, the paraoxonase 1 activity, cholesterol efflux rates, expression of ABCA1 and SR-BI in hepatocytes and peritoneal macrophages, and the level of ABCA1 and SR-BI in aortic lesions were remarkably improved in the probucol group, But the serum HDL cholesterol concentration, myeloperoxidase activity, the IMT and the percentage plaque area of aorta were significantly decreased.

**Conclusion:**

Probucol alleviated atherosclerosis by improving HDL function. The mechanisms include accelerating the process of reverse cholesterol transport, improving the anti-inflammatory and anti-oxidant functions.

## Background

Numerous epidemiological studies reported an inverse relationship between high density lipoprotein cholesterol (HDL-C) and the incidence of cardiovascular disease. The national cholesterol education program adult treatment panel III guidelines identified that low HDL-C (<40 mg/dl) is a major risk factor for coronary heart disease (CHD), independent of triglycerides and total cholesterol; for every 1 mg/dl increase in HDL-C, the predicted incidence of coronary events decreases by 2% in men and 3% in women [1,2].

However, the relationships between HDL and CHD risk are more complex beyond the serum HDL-C levels. The Milano people who carry the apolipoprotein A-I Milano mutant have low serum HDL-C levels but do not confer an increased cardiovascular risk [3]. Additionally, the torcetrapib, an inhibitor of potent cholesteryl ester transfer protein (CETP), markedly increased the serum HDL-C levels, but the risk of deaths and cardiac events had been increased simultaneously in patients receiving tocetrapib [4]. Hausenloy and his colleagues found that HDL isolated from patients with CHD was ineffective as an antioxidant, but paradoxically, appeared to be pro-oxidant [5]. Given this complexity, it is not surprising that a single assay of serum steady-state HDL-C levels does not necessarily correlate with HDL function. Structural modification and composition alteration of HDL may lead to HDL loss of normal biological function, even though HDL-C levels is normal which still failed to inhibit atherosclerosis.

Probucol is a unique cholesterol lowering drug with anti-oxidant and anti-inflammatory properties that decreases HDL-C levels [6]. Multivitamins and probucol (MVP) trial revealed that probucol reduces coronary restenosis after percutaneous transluminal coronary angioplasty [7]. And probucol observational study illuminating therapeutic impact on vascular events (POSITIVE) showed that probucol was useful in lowering the risk of cardiovascular events in secondary prevention in spite of causing a decrease in HDL-C levels [8]. Although probucol decreases HDL-C levels, it shows greatly controlled progression of atherosclerosis. We concluded that probucol may improve HDL function.

The major cardiovascular protective effects of HDL function may be attributed to its role in reverse cholesterol transport (RCT), anti-oxidant and anti-inflammation and so on [9]. ATP binding cassette transporter A1 (ABCA1) and scavenger receptor class B type I (SR-BI) play the key role in the process of RCT, high expression of ABCA1 and SR-BI can reflect the atheroprotective function of HDL [10]. So we examined whether probucol promoted RCT by up-regulating the expression of ACBA1 and SR-BI in peritoneal macrophages and hepatocytes. Paraoxonase 1 (PON1) and Myeloperoxidase (MPO) are enzymes closely associated with HDL anti-oxidant function. PON1 contributes to the anti-oxidant effects of HDL and its activity is inversely related to the risk of cardiovascular diseases [11]. MPO participates in HDL-oxidation and its activity is positive correlation with the risk of cardiovascular diseases [12]. And we considered probucol may improve HDL anti-oxidant function by affecting serum PON1 and MPO activity.

## Results

### Effects of probucol on serum lipid and body weight

There were no significant difference in serum lipid levels and body weight among the three groups at the baseline. At the end of the research, the results of the serum lipid suggested that hypercholesterolemia model was successfully established, serum triglyceride (TG), total cholesterol (TC), LDL-C and HDL-C in atherosclerosis group were significantly higher than control group. Serum lipids were also increased in probucol group as compared with control group, but TC, LDL-C and HDL-C in probucol group were significantly lower than atherosclerosis group. There was no doubt that probucol reduced HDL quantity. There was no significant difference in body weight among the three groups throughout the experiment (Table 1).

**Table 1:** Serum lipid and body weight profiles in three groups' rabbits.

	TG	TC	LDL-C	HDL-C	BW(kg)
***Control group***					
0 week	1.04 ± 0.19	1.85 ± 0.12	1.00 ± 0.20	0.47 ± 0.12	2.01 ± 0.08
12 week	1.02 ± 0.15	1.79 ± 0.21	1.09 ± 0.23	0.45 ± 0.12	2.77 ± 0.04
***Atherosclerosis group***					
0 week	1.10 ± 0.14	1.77 ± 0.15	0.99 ± 0.20	0.48 ± 0.12	2.07 ± 0.06
12 week	1.58 ± 0.25*	23.26 ± 3.30*	18.09 ± 4.04*	1.11 ± 0.09*	2.88 ± 0.08
***Probucol group***					
0 week	1.02 ± 0.15	1.76 ± 0.11	1.00 ± 0.15	0.43 ± 0.10	2.03 ± 0.04
12 week	1.60 ± 0.17	16.70 ± 0.70^#^	13.79 ± 2.01^#^	0.51 ± 0.06 ^#^	2.72 ± 0.21

TC, total cholesterol; LDL-C, low-density lipoprotein cholesterol; TG, triglyceride; HDL-C: high-density lipoprotein cholesterol; BW: body weight. Units for TC, TG, LDL-C and HDL-C are mmol/L and BW is kg. All values are presented as mean ± S.D. (n = 6).

* P < 0.05, compared to control group; ^# ^P < 0.05, compared to atherosclerosis group.

### Effects of probucol on restraining aortic atherosclerosis

**Figure 1:**
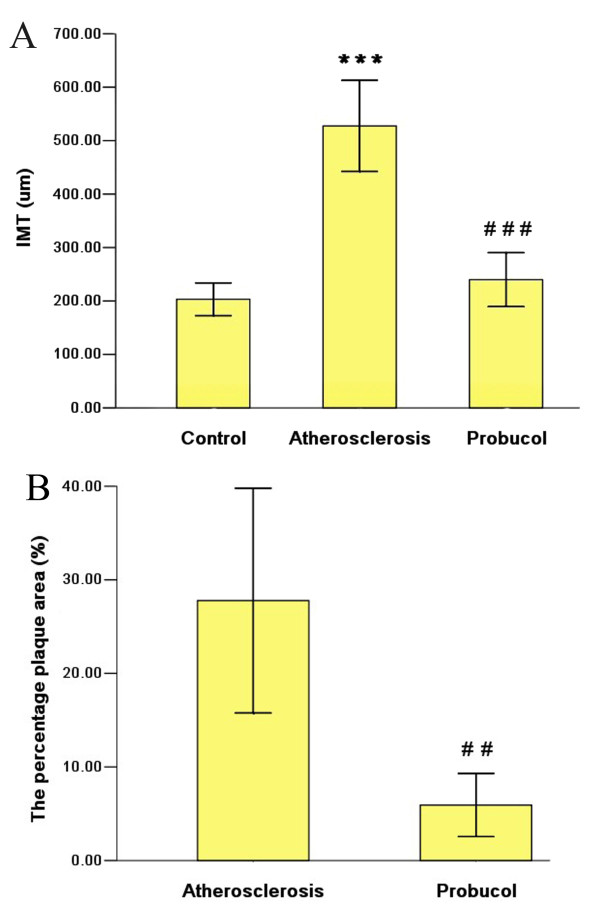
**The thoracic aortic IMT and the percentage plaque area (%) were decreased with the treatment of probucol**. *** P < 0.001, compared with control group; ^### ^P < 0.001, ^## ^P < 0.01 compared with atherosclerosis Group.

The thoracic aortic IMT and the percentage plaque area (surface area of plaque/surface area of whole intima) were significantly higher in the two cholesterol-fed groups than control group, which indicated that the expected atherosclerotic model were successful. Additionally, IMT and the percentage plaque area in probucol group were lower than atherosclerosis group, which revealed that probucol hindered the process of atherosclerosis (Figure 1).

### Effects of probucol on cholesterol efflux rate in hepatocytes and peritoneal macrophages

**Figure 2:**
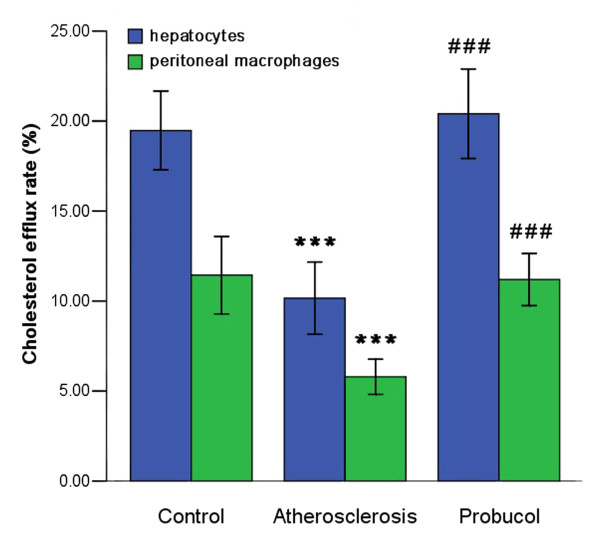
**Cholesterol efflux rate of hepatocytes and peritoneal macrophages were increased in probucol group**. *** P < 0.001, compared with control group; ^### ^P < 0.001, compared with atherosclerosis group.

We measured the HDL-induced cholesterol efflux rate in hepatocytes and peritoneal macrophages. The results showed that the cholesterol efflux rate of hepatocytes and peritoneal macrophages in atherosclerosis group were about half of control group. However, cholesterol efflux rate of hepatocytes and peritoneal macrophages in probucol group were about two-fold higher than atherosclerosis group. (Figure 2)

### ABCA1 or SR-BI mRNA and protein expressions in hepatocytes or peritoneal macrophages

**Figure 3:**
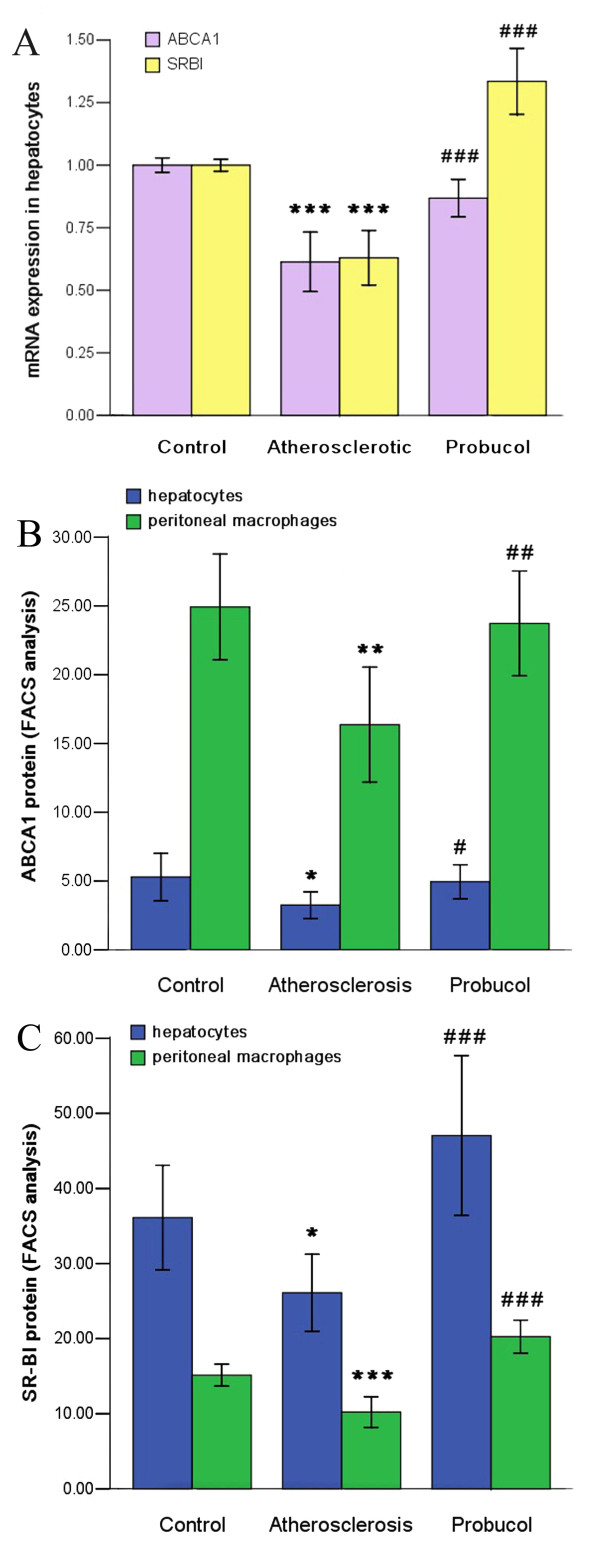
**Expressions of ABCA1 and SR-B1 were up-regulated by probucol**. (A) Hepatocytes ABCA1 and SR-B1 mRNA expression quantified by real-time RT-PCR. (B) ABCA1 protein expression of hepatocytes and peritoneal macrophages determined by flow cytometry. (C) SR-B1 protein expression of hepatocytes and peritoneal macrophages determined by flow cytometry.* P < 0.05, ** P < 0.01, *** P < 0.001 compared with control group; ^# ^P < 0.05, ^## ^P < 0.01, ^### ^P < 0.001 compared with atherosclerosis group.

We found that mRNA expressions of ABCA1 and SR-BI in hepatocytes were significantly lower in atherosclerosis group than in control group, but they were significantly higher in probucol group than in atherosclerosis group. Furthermore, the result also showed that mRNA expression of SR-BI was higher than ABCA1 in hepatocytes (Figure 3A).

At the same time, we measured ABCA1 and SR-BI protein expressions in hepatocytes and peritoneal macrophages. The results showed that ABCA1 and SR-BI protein expressions in probucol group both in hepatocytes and peritoneal macrophages were higher than in atherosclerosis group. More interesting, we found that protein expression of ABCA1 was higher than SR-BI in peritoneal macrophages, but expression of SR-BI was higher than ABCA1 in hepatocytes. Furthermore, we observed that as compare with atherosclerosis group, protein expressions of ABCA1 in hepatocytes and peritoneal macrophages on probucol group were increased by 44.96% and 52.15% respectively; and SR-BI in hepatocytes and peritoneal macrophages on probucol group were increased by 98.14% and 79.92%, respectively (Figure 3B-C).

### Immunohistochemical localization of ABCA1 and SR-BI in Aortic atherosclerosis lesions

**Figure 4:**
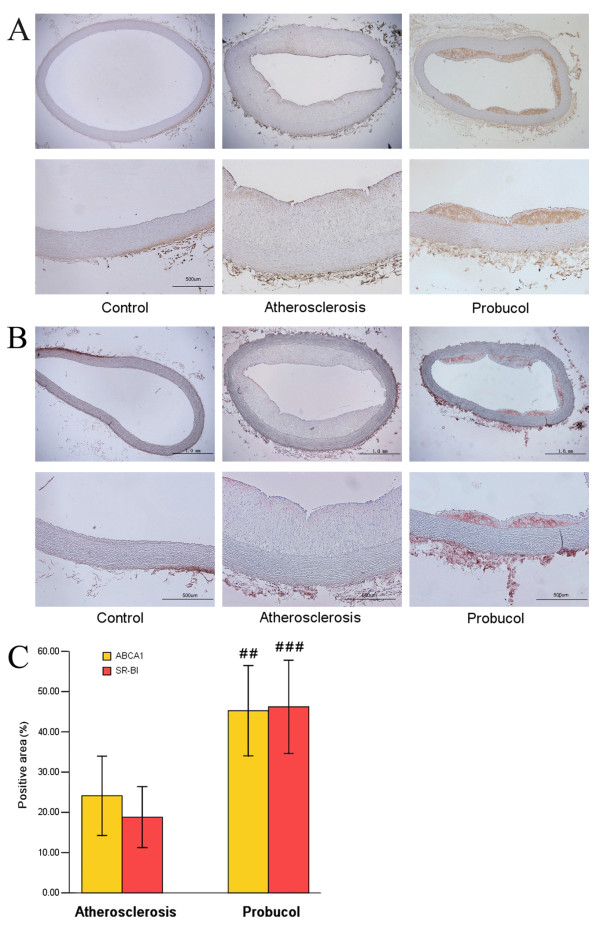
**Immunochemical staining of ABCA1 and SR-BI in aortic plaques**. Representative light microscopy images of aortic plaques (×40, below ×100). (A) ABCA1 staining (deep brown) in aortic plaque. (B) SR-BI staining (deep red) in aortic plaque. (C) ABCA1 and SR-BI staining positive area percentage were significantly increased in probucol group compared with atherosclerosis group. ^## ^P < 0.01, ^### ^P < 0.001 compared with atherosclerosis group.

In the control group, there were no atherosclerosis plaques and immunohistochemical staining in aortic walls for ABCA1 and SR-B1. So we just analyzed the percentage of the positive area in the two cholesterol-fed groups. As shown in the Figure 4, ABCA1 (deep brown) and SR-B1 (deep red) protein could be detected in aortic atherosclerosis plaques. We found that ABCA1 and SR-BI protein positive area were significantly higher in probucol group than in atherosclerosis group (Figure 4C).

### Effects of probucol on serum PON1 and MPO activity

**Figure 5:**
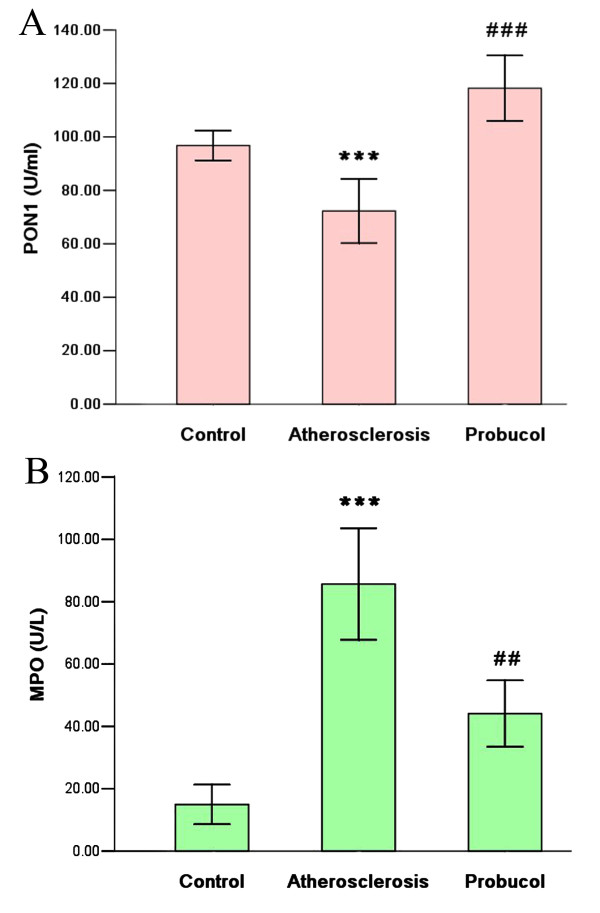
**Serum PON1 and MPO activity**. (A) Serum PON1 activity was significantly enhanced in probucol group as compared with atherosclerosis group. (B) Serum MPO activity was significantly reduced in probucol group as compared with atherosclerosis group. *** P < 0.001 compared with control group; ^## ^P < 0.01, ^### ^P < 0.001 compared with atherosclerosis group.

To study the effect of probucol on anti-oxidant and anti-inflammation properties of HDL, we detected the serum PON1 and MPO activity. Compared with the control group, the serum PON1 activity was extremely lower in atherosclerosis group, and serum PON1 activity was significantly increase when treated by probucol. In addition, extremely low MPO activity was found in the samples obtained from control group, and the MPO activity in atherosclerosis group showed significantly higher in comparison to the probucol group (Figure 5).

## Discussion

**Figure 6:**
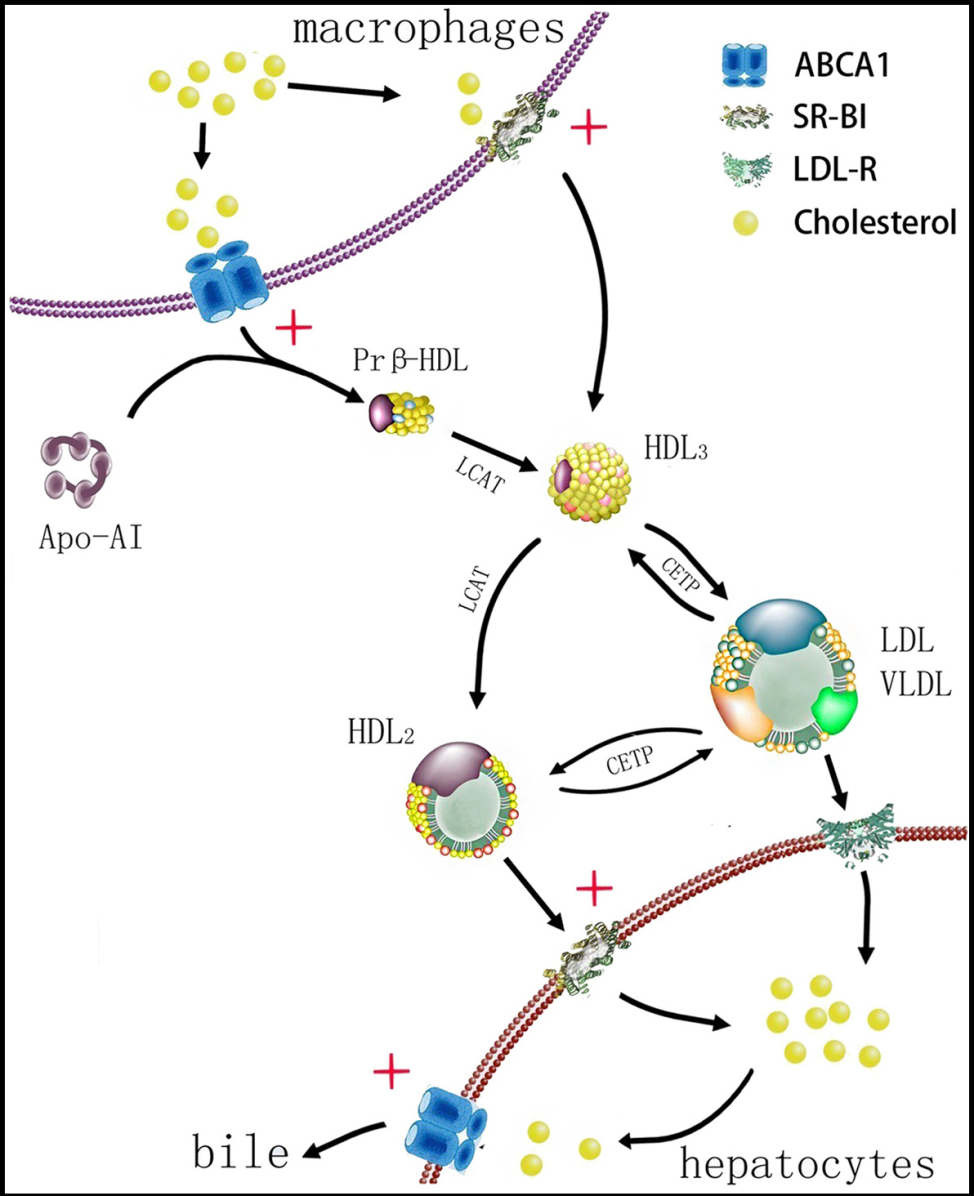
**Process of RCT and HDL metabolism**. Probucol up-regulate expression of ABCA1 and SR-B1 both in hepatocytes and peritoneal macrophages. (red+mean up-regulated the receptor expression)

The most important theory revolved around the role of HDL function is RCT, in which excess cholesterol effluxes to HDL and ultimately returns to the liver for metabolism. The process of RCT is extremely complicated: At first, cholesterol efflux from macrophages and then lipid-poor ApoA-I quickly acquires it via the ABCA1 transporter. Lipidation of the lipid-poor ApoA-I and cholesterol complex generates nascent (pre-β) HDL [13]. Subsequently, lecithin cholesterol acyl transferase (LCAT) mediated cholesterol esterification generates small HDL_3 _particles; small HDL_3 _can be converted to large mature HDL_2 _in turn upon CETP [14]. At last, these mature HDL_2 _transfer its cholesterol to the liver directly via SR-BI and excrete cholesterol through the bile (Figure 6) [15].

The best-recognized atheroprotective function of HDL is the promotion of cholesterol efflux from cells, particularly from macrophages and hepatocytes, because macrophages are the primary cell type to accumulate cholesterol within the atherosclerotic plaque and hepatocytes are the main cell type to excrete cholesterol through the bile [16]. In our research, we found that probucol increased the cholesterol efflux capacity of peritoneal macrophages, which could accelerate cholesterol effusing from macrophages that reduce cholesterol accumulating in artery wall. Additionally, probucol also increased the cholesterol efflux capacity of hepatocytes, which accelerated liver cells excreting cholesterol into the bile and decreased circulating lipids level.

ABCA1 is an efflux transporter for cholesterol and phospholipids, which is essential for the formation of HDL. Tangier disease patients and ABCA1^−/− ^mice fail to form discoid or spherical HDL, which exhibit abnormal lipid deposition in various tissues [17]. In macrophages, ABCA1 facilitates the transfer of the cholesterol to apoA-I, and then the lipidated apoA-I/ABCA1 complex dissociate and release the nascent HDL [18]. In our research, we found that probucol up-regulated the expression of ABCA1 in macrophages. It indicated that probucol accelerated cholesterol effusing from macrophages by ABCA1, which might promote further releasing of nascent HDL that has much more strong binding capacity to cholesterol. In the liver cells, highly expression of liver ABCA1 is to facilitate biliary excretion of cholesterol, which plays an important role in lipid homeostasis [19]. We found that probucol also promoted cholesterol excreting through the bile by ABCA1, which played an important role in reducing circulating cholesterol.

SR-BI also plays an important role in HDL metabolism. The catabolism of HDL is regulated in part by cholesterol ester removal or delipidation by SR-BI, which can controls the structure, composition, and concentration of HDL [20]. We found that probucol up-regulated the expression of SR-BI in macrophages and accelerate cholesterol effusing from macrophages. Rigotti and colleagues reported that they found abnormally large, heterogeneous, apoE-enriched, HDL like particles in SR-BI deficient mice, whose total serum cholesterol doubled [21]. Furthermore, the SR-BI deficiency greatly reduced the clearance of HDL cholesterol from serum [22]. Unfortunately, whether probucol reduce HDL-C levels by up-regulating expression of SR-BI in hepatocytes is unclearly. We found that probucol significantly increased expressions of SR-BI in hepatocytes. The increased SR-BI expression resulted in enhanced binding of large spherical HDL to the liver cells, which can speed up catabolism of HDL, and reduce serum HDL level. It may be the mechanism that probucol significant down-regulate HDL-C levels, but it didn't mean probucol declined HDL function, On the contrary it indicated probucol enhanced RCT and the metabolism of HDL.

HDL functions such as anti-oxidant and anti-inflammation are also important to restrain atherosclerosis. Native LDL has little effect on cells of the arterial wall, whereas oxidatively modified forms of LDL induce numerous proatherosclerotic effects [23]. HDL plays an important role in protecting against LDL oxidative modification, but dysfunctional HDL accumulates oxidants that promoting the formation of LDL-derived oxidized lipids and oxidation of LDL [24]. The enzyme PON1 plays the key role to the anti-oxidative effects of HDL. In our research, we found that rabbits treated by probucol have higher PON1 activity than atherosclerosis group. Navab and colleagues reported a failure of HDL to protect LDL from oxidation in patients with coronary atherosclerosis, which they proposed was due to their low serum PON1 activity despite relatively normal HDL-C levels [25]. PON1 can prevent lipid-peroxide accumulation on LDL and protect LDL from oxidative modification. Serum PON1 activity is lower in subjects prone to development of atherosclerosis such as in hypercholesterolemia disease [26]. Furthermore, some studies have proposed that PON1 may first associate with smaller HDL_3_, which are subsequently transformed into larger HDL_2 _particles. In this context, PON1 would be following the normal metabolic course of HDL in which remodelling leads to an increase in particle size, notably due to the accumulation of lipid components [27,28]. High PON1 activity may improve HDL anti-oxidant function.

Increasing evidences suggest that HDL is subjected to oxidation in lesion material that reverses the atheroprotective function [29]. In vitro studies demonstrated that MPO catalyzes oxidative modification of HDL leading to the development of dysfunctional proinflammatory and proatherogenic HDL [30]. MPO has been detected in human atherosclerotic lesions and high serum MPO is reported to be a risk factor for early adverse cardiac events in patients with acute coronary syndromes [31]. In addition, MPO was injected into mice led to significant reductions in RCT to the serum and fecal compartments, which demonstrating a direct effect of MPO on RCT in vivo[32]. In our research, we found probucol significantly decreased MPO activity, which in turn may reduce HDL oxidative modification and improve the process of RCT.

## Conclusions

In summary, probucol increased the expression of ABCA1 and SR-BI in macrophages and hepatocytes, which speeded up the process of RCT. Furthermore, probucol improved HDL anti-oxidant function by increasing serum PON1 activity. Probucol also reduced oxidative and inflammatory modification of HDL by decreasing serum MPO activity. Probucol reduced HDL-C level but improved HDL function and hindered the progression of atherosclerosis.

## Methods

### Animal model

Eighteen New Zealand white male rabbits (four months old, weighing 1.89 kg ~2.15 kg) provided by the Laboratory Animal Centre of Southern Medical University and individually housed in air-conditioned room. After a 7-day adaptation period, they were randomly divided into three groups: control group animals (n = 6) were fed a regular diet for 12 weeks; the atherosclerosis group (n = 6) were fed a high fat diet supplemented with 1% (w/w) cholesterol, 8% lard (w/w) and 0.05% cholate (w/w); and the probucol group were fed with the same high fat diet plus probucol (400 mg/ kg/day). Each rabbit consumed about 120 g of food daily. Rabbits were caged individually with water ad libitum for 12 weeks, and maintained on a 12-h day/night cycle. Animal procedures were performed in accordance with guidelines set by the Animal Experiment Committee of Southern Medical University Guangzhou, China.

### Serum lipid, PON1 and MPO activity analyses

Serum triglycerides (TG), total cholesterol (TC), low-density lipoprotein cholesterol (LDL-C) and HDL-C concentrations were measured with an automated biochemical analyzer (Type AU5421, Olympus, Japan) at the baseline and at the end of the study.

Serum PON1 activity was assayed with the method reported by Beltowski, using the synthetic substrate phenyl acetate (Sigma Co, American) [33]. PON1 activity towards phenyl acetate was determined by measuring the initial rate of substrate hydrolysis within the assay mixture (3 mL) containing 2 mM phenyl acetate, 2 mM CaCl_2_, and 10 μL of plasma in 100 mM Tris-HCl (pH 8.0). Absorbance was monitored for 90 seconds at 270 nm and enzyme activity was calculated from the E_270 _of phenyl acetate (1,310/M/cm) and expressed in U/mL (where 1 U of arylesterase hydrolyzes 1 μmol of phenyl acetate/min).

MPO activity was measured by a MPO determination kit (Jiancheng Bioengineering Co) using commercially available reagents, according to the manufacturer's instructions. Briefly, the serum samples were incubated in a 50 mM sodium phosphate buffer containing 1.5 M hydrogen peroxide and 0.167 mmol O-dianisidine dihydrochloride for 30 min. The increase in absorbance at 460 nm was recorded with the use of a spectrophotometer and the Enzymatic activity was calculated from E_460 _= 11300/M/cm. A unit of MPO activity is defined as the amount of enzyme degrading 1 μmol H_2_O_2 _per minute at 37°C.

### Isolation of hepatocytes and peritoneal macrophages

At the end of 12 weeks of the experiment, rabbits were anesthetized by 2% pentobarbital. Under sterile conditions, the parenchymal hepatocytes were isolated by classic *in situ *two steps perfusion of the liver with collagenase IV (0.05%) by enzyme digestion with collagenase II (2 mg/ml). The peritoneal macrophages were collected by peritoneal lavage with 200 ml PBS and purified by adherent method. Subsequently, using a modified method described by Zhao et al [34].

### Assay of HDL-mediated cholesterol efflux rate

Experiments were performed as previously described [35]. Rabbit peritoneal macrophages and hepatocytes were respectively seeded in medium with 0.2% BSA and antibiotics cultured to 2 × 10^5 ^cells/ml. Subsequently, culture medium was replaced for 24 h with a medium containing 0.2% BSA, 30 μg/ml Ox-LDL, and 1 μCi/ml [1α, 2α-^3^H]-cholesterol (Perkin-Elmer) in DMEM/F12. After cholesterol loading, cells were washed twice with serum-free medium. Cells were incubated in DMEM/F12 with cAMP (0.3 mmol/L) for the next 12 h. And then, apoA-I (10 ug/ml) was added to promote free cholesterol efflux for 4 h. the incubation medium was collected and centrifuged before counting radioactivity. The cell monolayer was washed with PBS and lysed with 1 ml of NaOH(0.1 mol/L). The radioactivity of medium and cell lysates was measured by liquid scintillation spectrometry (Beckman Instruments, Inc.). The cholesterol efflux rate was expressed as the medium [^3^H] cholesterol radioactivity as a percentage of total [^3^H] cholesterol radioactivity (cells plus medium). Individual efflux values were calculated as the averages of 3 determinations in different wells.

### Analyses of ABCA1 and SR-BI mRNA in liver by real-time PCR

Liver tissue was snap-frozen in liquid nitrogen until RNA extraction. Tissues were then powdered in liquid nitrogen. The quantitative analyses of ABCA1 and SR-B1 mRNA level on rabbit liver tissues were performed by quantitative real-time polymerase chain reaction (RT-PCR).Total RNA was extracted from the frozen liver tissues by using RNA Trizol reagent (Gibco RRL, Gaithersburg, USA). Then first-strand cDNA was synthesized by using PrimeScript RT Reagent Kit (Takara, China) according to the instruction from manufacture. The quantitative RT-PCR was performed on iQ5 Real Time PCR Detection System (Bio-Rad, American) using AllinOne™ Q-PCR Mix (GeneCopoeia Inc, American). Expression levels of the target genes generated standard curves were normalized against an endogenous reference gene glyceraldehydes 3-phosphate dehydrogenase (GAPDH). The specific primer sequences were listed in the following pattern: (1) ABCA**1: **forward primer: 5'-GAT GGC AAT CAT GGT CAA TGG -3', reverse primer: 5'-AGC TGG TAT TGT AGC ATG TTC CG-3' yielding a 201-bp size product; (2) SR-BI: forward primer: 5'-CAG TGG GCA TTG TGT CCT GTC -3', reverse primer: 5'-GGC TCA GTG CAG GCT GAT GTC-3' yielding a 286-bp size product; and (3) GAPDH: forward primer: 5'-GGA GCC AAA AGG GTC ATC-3', reverse primer: 5'-CCA GTG AGT TTC CCG TTC-3' yielding a 346-bp size product. PCRs for each sample were performed in duplicate, and the relative gene expressions were analyzed as described previously [36].

### Analyses of ABCA1 and SR-BI protein expression by flow cytometer

The protein assay of ABCA1 and SR-B1 in peritoneal macrophages and hepatocytes were performed as previously described by Pirillo et al [37]. The suspension of peritoneal macrophages and hepatocytes were respectively mixed with the antibody of ABCA1 or SR-B1(Santa Cruz Biontechnology, Inc) for 60 min at room temperature in the test tubes. The mixture was then washed two times with PBS and added the antibody labelled PE fluorescence (Seretec, Inc). After 30 min ABCA1/ SR-B1exprssion was measured by flow cytometer (BD FACSCalibur) and expressed as an average level in every hundred detected cells.

### Quantification of aortic atherosclerosis, histology and immunohistochemistry

All segments were embeded in paraffin and cut into 4-μm cross sections and stained with hematoxylin and eosin (H&E) for histological examination. The percentage plaque area, which was defined as surface area of plaque/surface area of whole intima, and the aortic intima-media thickness (IMT) were calculated for evaluating the degree of aortic atherosclerosis.

Experiments were performed as previously described [38]. Immunostaining for ABCA1 (Boster Biotechnology Co. Ltd China) and SR-BI (Abcam Co.Ltd American) was performed in paraffin-embedded Aortic atherosclerosis sections using the specific antibody and a Strept Avidin-Biotin Complex (SABC) Antibody binding was visualized with SABC kits (Boster Biotechnology Co. Ltd), Diaminobenzidine (DAB) and 3-Amino-9-Ethylcarbazole (AEC) were used as the chromogen and Mayer's hematoxylin as the nuclear counterstain. The sections were dehydrated, cleared, mounted and performed by morphometric analysis. All sections (H&E and immunostained) for microscopic quantification were captured under an Olympus BX51 light microscope equipped with a DP70 digital camera (Olympus, Tokyo, Japan) and were measured with Image pro plus 6.0 special image analysis software (Media Cybernetics Co, American).

### Statistical analysis

Data are presented as mean ± S.E.M. A one-way ANOVA was used for analyzing differences in variables between groups at the same time point. When P was ≤ 0.05, the least significant difference method was used for comparison. An independent sample t-test was used for analyzing the differences in variables between two groups at the same time point. SPSS 13.0 software was used for statistical analysis with P < 0.05 indicating a statistically significant difference.

## Abbreviations

**HDL-C : ***High density lipoprotein cholesterol*; **LDL-**C *Low-density lipoprotein cholesterol*; **VLDL : ***Low-density lipoprotein cholesterol *; **TC : ***total cholesterol*; **TG : ***Triglyceride*; **PON1 : ***Paraoxonase *1; **MPO : ***Myeloperoxidase*; **RT-PCR : ***Real-Time Reverse transcription polymerase chain reaction*; **FACS : ***flow cytometer*; **ABCA1 : ***ATP binding cassette transporter A1*; **SR-BI : ***scavenger receptor class B type I*; **IMT : ***intima media thickness*; **CHD : ***Cardiovascular heart disease*; **Apo-AI : ***apolipoprotein-AI*; **CETP : ***cholesteryl ester transfer protein*; **LCAT : ***lecithin cholesterol acyl transferase*; **RCT : ***reverse cholesterol transport*,.

## Competing interests

The authors declare that they have no competing interests.

## Authors' contributions

ZGG was responsible for the experimental design, supervising the project, data analysis and revising the manuscript. JKZ carried out all aspects of experiments, data analysis and drafted the manuscript. CL was involved in pathohistology, immunoassays and all samples collected. ZKW participated in Constructed Animal models, collected blood sample, assayed cholesterol efflux rate and RT-PCR. YWL, YT were involved in Cell culture, flow cytometer and supervising the project. All authors read and approved the final manuscript.
